# Validation of portable electronic equipment (Accutrend^®^ Plus) to determine glucose, total cholesterol, and triglycerides in rats (*Rattus*) and dogs (*Canis lupus familiaris*)

**DOI:** 10.5455/javar.2023.j652

**Published:** 2023-03-31

**Authors:** Paola de la Paz Ramírez, Gerardo Ordaz, Reynaldo de la Paz Gonzáles, Rosa Elena Pérez, Manuel López, Ruy Ortiz

**Affiliations:** 1 Facultad de Medicina, Veterinaria y Zootecnia, Universidad Michoacana de San Nicolas de Hidalgo, Michoacán, México; 2 Centro Nacional de Investigación Disciplinaria en Fisiología y Mejoramiento Animal-INIFAP, Querétaro, México; 3 Facultad de Ciencias Médicas y Bilógicas Dr. “Ignacio Chávez,” Universidad Michoacana de San Nicolas de Hidalgo, Michoacán, México; 4 Facultad de Químico Farmacobiología, Universidad Michoacana de San Nicolas de Hidalgo, Michoacán, México

**Keywords:** Blood chemistry, concordance, linearity, correlation

## Abstract

**Objective:**

This study aimed to validate the analytical precision of the Accutrend^®^ Plus portable electronic equipment (PE) to determine glucose (GLU), total cholesterol (TC), and triglycerides (TG) in rats and dogs using the conventional laboratory method (CM) as a reference.

**Materials and Methods:**

To determine the analytical accuracy of the Accutrend^®^ Plus in the measurement of GLU, CT, and TG. The EP-9-A2 guide (Clinical and Laboratory Standards Institute), Bland-Altman graphical analysis, and Lin’s correlation coefficient of concordance (CCC) were implemented.

**Results:**

The average differences (*p* > 0.05) between PE and CM for GLU, TC, and TG were 2.21, 1.20, and 0.72 mg·dl^−1^, respectively, in rats and 1.06, 4.30, and 2.41 mg·dl^−1^, respectively, in dogs (*p* > 0.05). Both methods showed a linear relationship with Pearson’s correlation coefficients > 0.96 and *R*^2^ > 0.97 for the three biochemical indicators evaluated in both species. The GLU, TC, and TG values obtained by the PE were substantial, as evident from Lin’s CCC > 0.96.

**Conclusion:**

The PE Accutrend^®^ Plus is potent for monitoring GLU, TC, and TG in rats and dogs because of its precision and ability to facilitate measurement by reducing stress in animals during sampling.

## Introduction

Animal welfare is imperative in research studies using animals as biological models because, like humans, animals react to various environmental stimuli. In addition, it is important to apply the principles of substitution, reduction, and refinement in research using animal models (3Rs) [1]. Using animals as biological models helps a more precise understanding of physiological and pathological processes (control, treatment, and prevention) in humans and animals [2]. In addition, animal models promote better testing of the efficacy and safety of drugs and new chemical and biological agents before their applications in humans and animals [3].

The epistemological foundation for using animals as experimental models is based on the morpho-physiological characteristics of animals and their similarities with human physiology [4]. However, there is experimental research on phenomena inherent to the animal species with which it is experimented [5]. How animals are subjected to experimentation to answer scientific questions is the real problem of animal experimentation [4]. The exposure of animals to any type of risk or harm cannot be justified unless the following criteria are met [6]: I) the research must have social value; II) the protocols must have scientific validity; III) the risk-benefit ratio must be favorable, ensuring the maximum welfare for the animal and causing the minimum amount of pain and suffering; and IV) the handling of the animals under experimentation must be carried out by competent personnel [7]. Percie et al. [8] established that “an investigation that has no scientific validity is not ethical.” The potential usefulness of the results is a prerequisite to justifying the use of animals in experiments. It is not simply a matter of using a few animals; it is a matter of using as few as possible, but considering that this number must guarantee that the results can be analyzed with statistical rigor and allow a valid result to be reached. It is as bad to use animals in excess as the number is scarce because if the experiment is not conclusive, then in this case, all the experimental animals will have been used uselessly.”

Since animal research is conducted to solve problems inherent to animal species, it may be essential to design experiments that warrant repeated measurements of samples to determine biochemical indicators associated with the physiology or pathology under study [9,10]. This may stress animals because obtaining samples may require physical or chemical containment. The containment process, however subtle, involves time and practices of immobilization and manipulation of the animal [11,12]. This management is not routine, nor does it belong to a “natural” management process in the human-animal relationship. Thus, it inevitably causes unnecessary stress and pain in animals during experimentation or those with a disease that requires diagnosis or evolutionary monitoring of certain biochemical indicators [7]. This is particularly the case with the determination of glucose (GLU), total cholesterol (TC), and triglycerides (TG) in animals as biological models or in clinical practice for the diagnosis of pathological processes. Indicators in human clinical practice are currently determined quickly using portable electronic equipment (PE) such as Accutrend^®^ Plus. However, it is unknown whether this device has the precision to be implemented in rats (*Rattus norvegicus*), a universal biological model for research, and dogs (*Canis familiaris*), a species with the greatest diffusion in veterinary clinical practice. Following the above, the objective of this study was to validate the Accutrend^®^ Plus PE to determine GLU, TC, and TG in rats and dogs.

## Materials and Methods

The research was carried out in the bioterio and in the Auxiliary Diagnostic Services Unit located in the Posta Zootécnica of the Faculty of Veterinary Medicine and Zootechnics (FVMZ) of the Universidad Michoacana de San Nicolás de Hidalgo (UMSNH). The protocol was reviewed and approved by the Technical Scientific Committee of the FVMZ-UMSNH. Animal handling was conducted according to the guidelines of the Official Mexican Standard for the Production, Care, and Use of Laboratory Animals [13], as well as those of the International Guiding Principles for Biomedical Research Involving Animals [14].

### Animals and accommodation

Twenty rats and 20 dogs were used. Adult rats (10 ♀ and 10 ♂) with an average weight of 136.5 ± 0.5 ♀ and 215 ± 0.5 gm belonged to the FMVZ-UMSNH bioterio; clinically healthy adult Beagle breed dogs (10 ♀ and 10 ♂) with an average weight of 20.2 ± 3.5 kg ♀ and 18.1 ± 2.02 kg ♂ were obtained through routine consultations in private veterinary clinics in Mexico. The selection, handling, and sampling of the dogs were carried out with the prior informed consent of the owners. 

The rats were housed in individual cages (33 × 23 × 15 cm, length, width, and height, respectively) made of acrylic (6 mm thick) with a steel grid-type lid, with sections for food and water. A commercial feed (Purina^®^) was provided to rodents *ad libitum*. Drinking water was freely accessible in plastic troughs with glass tips. The dogs used in this study were companion animals housed indoors and fed species-specific commercial food. The drinking water was freely accessible to these animals.

### Sampling and biochemical indicator determination

The variables evaluated were GLU, TC, and TG. Biochemical indicators were determined by the PE Accutrend^®^ Plus, Roche, and by the conventional enzymatic/colorimetric method (CM).

Blood samples (0.3 ml·rat^−1^) from rats were obtained from the tail vein, and the blood volume was deposited in a vacutainer^®^ tube without a coagulation activator and with separating gel. Samples (5.0 ml·dog^−1^) in dogs were obtained from the cephalic vein and stored in a vacutainer^®^ tube without a coagulation activator and with separating gel; preprandial samples were obtained in both species. Before sending the samples to the laboratory for storage and analysis, a drop of blood (0.5 µl) was extracted from each sample obtained from rats and dogs and placed on specific reagent strips for Accutrend^®^ for the determination of GLU, TC, and TG. In the laboratory, samples from rats and dogs were centrifuged at 1,000 × g for 10 min for serum extraction. The serum was placed in 1.5 ml Eppendorf^®^ tubes, and GLU, TC, and TG levels were subsequently determined using an automated enzymatic/colorimetric CM, adapted to a Cobas^®^ c111Mira (Roche, Basel, Switzerland MR).

### Statistical analysis

The information collected by both methods (CM and PE) was statistically analyzed under the criteria of the Clinical and Laboratory Standards Institute (CLSI) [15], section EP-9-A2, as follows: 1) To check the normality of the parameters studied, the Kolmogorov-Smirnov statistical test was used. 2) To determine the extreme values (*outliers*), absolute differences between the methods were compared. Said differences must not exceed four times the value of the mean of the absolute differences. 3) To estimate the correlation coefficient (*r*), the interval of values was considered adequate if the *r *value was greater than or equal to 0.950; therefore, a linear regression was used to estimate the slope and the ordinate at the origin. 4) Through linear regression, the values of the slope and the ordinate to the origin were obtained for each pair of results along with their respective 95% confidence intervals. 5). Estimating the systematic error and the CI of the tested method was based on the clinical decision levels for GLU, TC, and TG from the linear regression equation.

Once the five criteria indicated in the previous paragraph were fulfilled, the concordance between both methods (CM *vs.* PE) was evaluated using Bland-Altman graphical analysis and Lin’s correlation coefficient of concordance (CCC). For these analyses, the statistical package SAS^®^ (SAS 9.4 Inst. Inc., Cary, NC) software was used.

## Results

No difference (*p* > 0.05) was found between the mean GLU, TC, and TG values obtained by the PE (Accutrend^®^ Plus) and laboratory CM (Cobas c111^®^) in rats and dogs (Table 1). In rats, the confidence intervals for CM and PE were 96.9–110.2 and 99.1–113.1 mg·dl^−1^ for GLU, 46.7–54.5 and 46.9–56.5 mg·dl^−1^ for TC, and 89.1–115.5 and 89.8–115.4 mg·dl^−1^ for TG, respectively. In dogs, the confidence intervals for CM and PE were 86.7–102.2 and 85.8–99.9 mg·dl^−1 ^for GLU, 174.3–210.5 and 169.4–206.8 mg·dl^−1^ for TC, and 95.6–144.1 and 97.4–147.0 mg·dl^−1^ for TG, respectively (Table 1).

In rats, the coefficients of variation of the differences between both methods were 2.8%, 3.1%, and 4.4% for GLU, TC, and TG, respectively. In dogs, the coefficients of variation were 2.1% for GLU, 2.6% for CT, and 4.4% for TG (Table 1). In rats, the average difference in the values obtained by both methods (average of the three indicators evaluated) was 3.9 mg·dl^−1^ for minimum values, 4.1 mg·dl^−1^ for maximum values, and 1.3 mg·dl^−1^ for the arithmetic mean. In dogs, the average difference was 0.9 mg·dl^−1^ for minimum values, 2.9 mg·dl^−1^ for maximum values, and 2.6 mg·dl^−1^ for the arithmetic mean.

The results of the statistical analysis (Kolmogorov-Smirnov) revealed the normal distribution of the GLU, TC, and TG values obtained by the CM and PE both in rats and dogs (*p* > 0.05) (Table 1). Furthermore, there were no outliers that were greater than one standard deviation. Regarding linearity and constant dispersion, the two methods presented a linear relationship because the Pearson correlation coefficient was >0.96 for each evaluated indicator in rats and dogs (Table 2).

**Figure 1.**
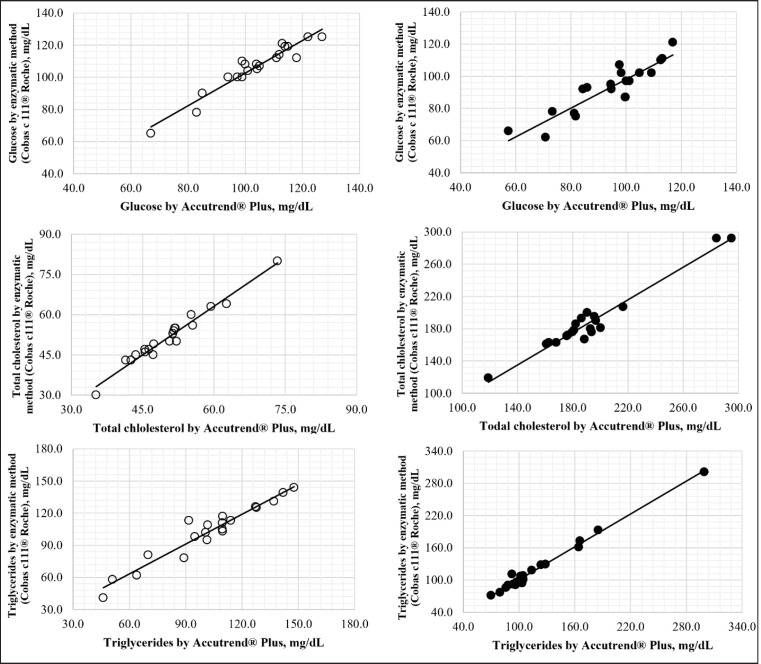
Linear estimation between enzymatic method (Cobas c111^®^ Roche) and Accutrend^®^ Plus PE for GLU, TC, and TGs in rats (°) and dogs (●).

The determination coefficients were higher than 0.90 and showed a constant dispersion; hence, linear regression was used to check the linearity of the measurement techniques studied (Fig. 1). The regression equations for each indicator evaluated for each species are listed in Table 2. Lin’s CCC was > 0.96 for each indicator, both in rats and dogs (Table 2).

**Figure 2.**
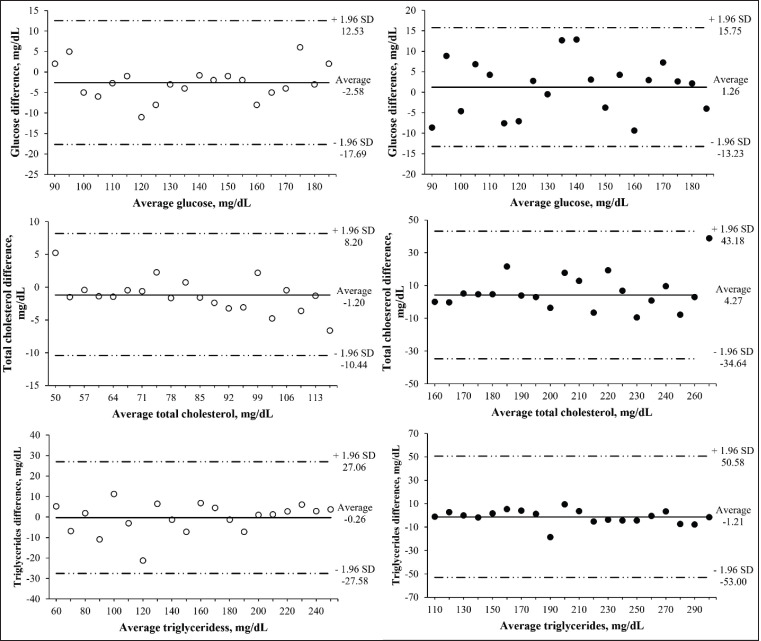
Bland-Altman plots between enzymatic method (Cobas c111^®^ Roche) and Accutrend^®^ Plus PE for GLU, TC , and TGs in rats (°) and dogs (●).

According to the Bland-Altman graphical method for the indicators evaluated in rats, the systematic bias and limit of concordance were −2.58 mg·dl^−1^ and between −17.69 and 12.53 mg·dl^−1^ for GLU, −1.20 mg·dl^−1^ and between −10.44 and 8.20 mg·dl^−1^ for TC, and −0.26 mg·dl^−1^ and between −27.58 and 27.06 mg·dl^−1^ for TG (Fig. 2). Regarding the indicators evaluated in dogs, the systemic bias was 1.26, 4.27, and −1.21 mg·dl^−1^ for GLU, TC, and TG, respectively, and the limit of concordance was between −13.23 and 15.75 mg·dl^−1^ for GLU, −34.64 and 43.18 mg·dl^−1^ for TC, and −53.00 and 50.58 mg·dl^−1^ for TG (Fig. 2). In both rats and dogs, there were no values outside the limits (differences at ±1.96 standard deviations) for the evaluated indicators (Fig. 2).

## Discussion

The restructuring of the ethics guidelines for the use of animals in research has led to the strict implementation of animal welfare regulations; therefore, research with animals as objects of study must seek new strategies to limit their use or minimize their stress during experimental processes. Therefore, the validation of less invasive procedures is vital to smoothly implementing the use of animals in research.

The EP-9-A2 guide was implemented for the validation of the Accutrend^®^ Plus. The procedures used in this guide do not correspond to the classical statistical validation methods; however, as it is an effective validation method, it has been proposed as a validation tool for both accredited laboratories with flexible scope and for validation of certified laboratory methods [16].

We established the differences between the means of both methods in rats and dogs for GLU (21.4% and 1.1%), TC (2.3% and 1.2%), and TG (0.3% and 2.0%) (Table 1). For quality specifications, the CLSI (2021) established that the values obtained by alternative methods should not exceed ±10% of variability for GLU and TC and ± 25% for TG in relation to the values reported by the reference method. Therefore, the PE (Accutrend^®^ Plus; (Table 1) meets the quality criteria stipulated by the CLSI. This observation is in line with that reported by Skeie et al. [17], who established that the maximum value of the accepted coefficient of variation to validate a reliable alternative method is 5%. In addition, the variation between both analyzed methods was <4.4% for each evaluated indicator in rats and dogs (Table 1).

**Table 1.** Descriptive results of GLU, TC, and TGs (mg·dl^−1^) in rats and dogs. Values obtained through PE (Accutrend^®^ Plus) and conventional laboratory method (CM, Cobas c111^®^ Roche).

Rats						
	GLU		TC		TGs	
**Indicated**	**CM**	**Accutrend** ^®^	**CM**	**Accutrend** ^®^	**CM**	**Accutrend** ^®^
Minimum value	67.01	65.71	35.22	30.00	46.18	41.00
Maximum value	127.11	125.13	73.39	80.24	147.70	144.01
Arithmetic average	103.53 ± 14.23	106.10 ± 15.01	50.55 ± 8.33	51.75 ± 10.25	102.31 ± 28.18	102.60 ± 27.16
Mean 95% CI	96.86 ± 10.82	99.07 ± 11.41	46.65 ± 6.34	46.95 ± 7.79	89.11 ± 21.43	89.83 ± 20.65
	110.20 ± 20.79	113.10 ± 21.92	54.45 ± 12.17	56.55 ± 14.97	115.50 ± 41.16	115.39 ± 39.67
Normality test^a^	0.1309	0.1922	0.1723	0.1467	0.1271	0.1419
	**Variation coefficients, %**					
Minimum	0.54		0.57		0.67	
Maximum	7.44		11.32		16.63	
Average	2.79		3.12		4.44	
**Dogs**						
	**GLU**		**TC**		**TGs **	
**Indicated**	**CM**	**Accutrend** ^®^	**CM**	**Accutrend** ^®^	**CM**	**Accutrend** ^®^
Minimum value	57.38	56.00	118.90	119.00	69.99	71.13
Maximum value	117.00	121.00	295.12	292.00	299.40	301.00
Arithmetic average	93.91 ± 15.48	92.85 ± 15.10	192.40 ± 38.68	188.10 ± 40.03	119.80 ± 51.81	122.21 ± 53.09
Mean 95% CI	86.66 ± 11.77	85.78 ± 11.48	174.30 ± 29.41	169.40 ± 30.44	95.58 ± 39.40	97.35 ± 40.37
	102.20 ± 22.61	99.92 ± 22.06	210.50 ± 56.49	206.81 ± 58.47	144.10 ± 75.67	147.00 ± 77.54
Normality test^a^	0.1653	0.1330	0.2688	0.2334	0.2649	0.2403
**Variation coefficients, %**						
Minimum	0.35		0.04		0.04	
Maximum	4.84		8.59		12.94	
Average	2.14		2.62		4.35	

^a ^Kolmogorov-Smirnov.

The statistical analysis (Kolmogorov-Smirnov) and the linear distribution revealed a constant dispersion (*r* > 0.96; *p* < 0.05) between both methods for each metabolite evaluated in dogs and rats (Table 1 and 2; Fig. 1). Thus, the alternative method (Accutrend^®^ Plus) maintained the ratio between the concentration of the analyte and its response [18]. It has been established [19] that Pearson’s correlation coefficient must be greater than 0.95 for a calibration curve, although a value equal to 0.95 is sometimes acceptable. However, there are controversies about implementing correlation coefficients to determine linearity when validating a method [18,19]. Morón et al. [18] reported that the best indicator to establish linearity in the validation of an analytical method was calculated by the Student’s t-test for Pearson’s correlation coefficient (*tr*) with *n*−2 degrees of freedom. The results showed that *tr* was significant (*p* = 0.05).

In contrast to the *tr* method, Lin’s CCC is a coefficient that qualifies the strength of association as almost perfect for values greater than 0.99, substantial for values from 0.95 to 0.99, moderate for values from 0.90 to 0.94, and poor for values below 0.90 [20,21]. In this study, Lin’s CCC was >0.96 for the evaluated indicators in dogs and rats; thus, GLU, TC, and TG determinations in rats and dogs obtained by Accutrend^® ^Plus were within the category of substantial agreement. Thus, PE Accutrend^®^ Plus is viable as an alternative method for measuring GLU, TC, and TG in both species tested if the results (*r*, *R*^2^, *tr*, and Lin’s CCC) are considered. Once the correlation criteria described above are fulfilled, it is necessary to use the linear regression coefficients (β0 and β1; Table 2) to check the linearity of the measurement techniques being evaluated [18].

The agreement presented by the statistical analyses described above was determined using the Bland-Altman graphical method (Fig. 2), thus allowing evaluation of whether the difference between the values found has any relevance from a clinical point of view [22]. The results of the Bland-Altman graphical method did not show values outside the limits (differences at ±1.96 standard deviations) for each evaluated indicator in both species. The difference between PE and CM was constant at all concentrations of GLU, TC, and TG in both species according to the Bland-Altman analysis (Fig. 2). The minimal differences (*p* > 0.05) observed between the PE and CM were probably owing to the strips implemented in the PE that were calibrated to determine whole blood metabolites. In contrast, the indicators (GLU, TC, and TG) were determined in the serum, which may present an acceptable variation of ≤15% [15,23,24].

**Table 2.** Pearson’s correlation coefficient (*r*), linear regression equation, determination coefficient (*R*^2^), and Lin’s CCC between Accutrend^®^ Plus PE and enzymatic method (Cobas c111^®^ Roche) for GLU, TC , and TGs in rats and dogs.

RATS				
Indicator	*r*	Regression equation	*R* ^2^	Lin’s CCC
GLU	0.961	*Y*=6.941+0.910*X	0.92	0.993
TC	0.982	*Y*=9.243+0.798*X	0.96	0.970
TGs	0.964	*Y*=-0.278+1.001*X	0.93	0.963
**DOGS**				
GLU	0.981	*Y*=0.597+1.004*X	0.96	0.985
TC	0.977	*Y*=14.836+0.944*X	0.95	0.995
TGs	0.986	*Y*=2.209+0.962*X	0.97	0.989

## Conclusion

The Accutrend^®^ Plus PE is a viable device for monitoring GLU, TC, and TG in rats and dogs because of its precision and the ability to facilitate measurement by reducing stress in animals during sampling.

## List of abbreviations

PE, portable equipment; CM, conventional method; GLU, glucose; TC, total cholesterol; TG, triglycerides; CCC, correlation coefficient of concordance; CLSI, Clinical and Laboratory Standards Institute.
